# Cell Surface Cdc37 Participates in Extracellular HSP90 Mediated Cancer Cell Invasion

**DOI:** 10.1371/journal.pone.0042722

**Published:** 2012-08-17

**Authors:** Avraam El Hamidieh, Nicholas Grammatikakis, Evangelia Patsavoudi

**Affiliations:** 1 Department of Biochemistry, Hellenic Pasteur Institute, Athens, Greece; 2 Institute of Biology, National Center for Scientific Research “Demokritos”, Athens, Greece; 3 Department of Biomedical Instrumentation Technology, Technological Educational Institute of Athens, Athens, Greece; Institute of Molecular and Cell Biology, Singapore

## Abstract

Cdc37 is a 50 kDa molecular chaperone which targets intrinsically unstable protein kinases to the molecular chaperone HSP90. It is also an over-expressed oncoprotein that mediates carcinogenesis and maintenance of the malignant phenotype by stabilizing the compromised structures of mutant and/or over-expressed oncogenic kinases. Here we report that Cdc37 is not restricted intracellularly but instead it is also present on the surface of MDA-MB-453 and MDA-MB-231 human breast cancer cells, where it is shown to participate in cancer cell motility processes. Furthermore, we demonstrate using an anti-Cdc37 cell impermeable antibody, that similarly to its intracellular counterpart, this surface pool of Cdc37 specifically interacts with HSP90 as well as the kinase receptors HER2 and EGFR on the cell surface, probably acting as a co-factor in HSP90's extracellular chaperoning activities. Finally, we show that functional inhibition of surface HSP90 using mAb 4C5, a cell impermeable monoclonal antibody against this protein, leads not only to disruption of the Cdc37/HSP90 complex but also to inhibition of the Cdc37/ErbB receptors complexes. These results support an essential role for surface Cdc37 in concert with HSP90 on the cell surface during cancer cell invasion processes and strengthen the therapeutic potential of mAb 4C5 for the treatment of cancer.

## Introduction

HSP90 is a molecular chaperone that functions in association with a cohort of co-chaperones to guide the stabilization and activation of an array of signaling proteins, including oncogenic kinases, transcription factors and hormone receptors [1], [2]. Cdc37 (Cell division cycle protein 37) is considered as a key component of this multimeric chaperone machinery, playing a specialized and indispensable role in the maturation and/or stabilization of a large subset of protein kinases, implicated in signal transduction, proliferation and survival [3]. By blocking the closure of the N-terminal HSP90 ATP-binding site, Cdc37 inhibits the ATPase activity of HSP90 [4] and assists loading of kinase client proteins onto the chaperone machinery [4], [5]. Ιn particular, Cdc37 acts as an adaptor or scaffold, facilitating client kinase interaction with HSP90 [6] and subsequently by recruiting these client kinases into the HSP90 complex, it stabilizes and/or maintains them in a folding-competent conformation [7]. In addition, Cdc37 promotes the assembly of HSP90-protein kinase complexes [8] and expression of a dominant-negative form that lacks the HSP90-binding domain inhibits kinase activation in mammalian cells [9]. Many client proteins interact directly with both Cdc37 and HSP90 and their folding, maturation and stability depend on the activity of both chaperones. Hence Cdc37 mediates the formation of HSP90-Raf1 [9] and HSP90-Cdk4 complexes [10] and these interactions are necessary for protein stability and kinase function. The complex relationship between Cdc37 and HSP90 is illustrated by the finding that their interaction is stabilized by the client protein [11]. Over the past years there has been increasing evidence demonstrating that intracellular HSP90 plays a pivotal role in the acquisition and maintenance of the malignant phenotype [12], [13], [14], [15]. Accordingly, there is growing interest in Cdc37 in the context of malignancy [16], [17] since Cdc37 also regulates multiple oncogenic kinase clients. Indeed Cdc37 levels are found increased in many clinical cancers [17]. In particular, Cdc37, is increased in proliferating tissues, and is heavily expressed in certain cancers including anaplastic large cell lymphoma [18] acute myelocytic leukaemia [19], hepatocellular carcinoma [20] and multiple myeloma [21]. Furthermore, data have been presented indicating that Cdc37 can function as an oncogene, as mice engineered to over-express Cdc37 develop tumors at a high frequency [22], suggesting that the establishment of protein kinase pathways mediated by HSP90/Cdc37 can be a rate-limiting event in epithelial cell transformation [22]. More recently it has been shown that Cdc37 is essential for maintaining prostate tumor cell growth [23]. Additionally, the platelet-derived growth factor receptor alpha which is up-regulated and activated in several malignancies forms a complex with HSP90 and the co-chaperone Cdc37 in ovarian, glioblastoma, and lung cancer cells [24]. Together, these results support the targeting of Cdc37 for cancer therapy.

We and others have previously identified an extracellular pool of HSP90 both in normal and cancer cells which was shown to be involved in migration and invasion processes, respectively [25], [26], [27], [28], [29], [30]. Furthermore, and by exploiting the function blocking properties of a cell-impermeable monoclonal antibody named mAb 4C5, specifically targeted against HSP90 we have shown that extracellular HSP90 interacts with HER-2 on the cell surface [28] as well as metalloproteinases MMP-2 and MMP-9 [29]. Although a growing number of HSP90's co-chaperones such as HSP70, Hop and p23 were also found on the cell surface [31], [32], [33], their action and underlying mechanisms have not been elucidated yet. Taking the above into consideration, in the present work we explore the cell surface localization of Cdc37 and we examine its possible involvement in cancer cell invasion processes as well as its potential interacting partners during this process, using the MDA-MB-453 and MDA-MB-231 human breast cancer cell lines and a commercially available polyclonal antibody against Cdc37. Furthermore and taking into account previously reported data showing that mAb 4C5 inhibits cancer cell invasion by disrupting association of extracellular HSP90 with HER2 [28] and metalloproteinases MMP2 and MMP9 [29] in this work we investigate the possible effect of this cell impermeable anti-HSP90 antibody on the interactions of surface Cdc37 with HSP90 and the ErbB receptors.

## Materials and Methods

### Antibodies and reagents

MAb 4C5 was produced in our laboratory, as previously described [34]. In the present study, mAb 4C5 was used as concentrated serum-free supernatant in all experiments performed. Polyclonal antibodies against EGFR, HER-2 and monoclonal antibody against Cyclin D1 were obtained from Santa Cruz Biotechnology, Inc. (Santa Cruz, CA). Two anti-Cdc37 antibodies, from Santa Cruz Biotechnology and Abcam recognizing different epitopes were used. Polyclonal antibodies against HSP90 were from Chemicon International. DMEM, RPMI and fetal bovine serum (FBS), were from Invitrogen. All other materials were from sources previously described [25].

### Cell cultures and immunofluoresence

HER-2-over-expressing MDA-MB-453, EGFR-over-expressing MDA-MB-231 breast cancer cell lines and non cancerous epithelial breast cells MCF-12A were purchased from American Type Culture Collection. MDA-MB-453 and MDA-MB-231 cells were maintained in RPMI and DMEM, respectively, supplemented with 10% FBS. MCF-12A cells were maintained in DMEM-HAM F12 supplemented with 20 ng/ml human EGF, 100 ng/ml hydrocortisone, 0.01 mg/ml bovine insulin and 10%FBS.

For immunofluorescence studies, cells were plated on poly-L-lysine-coated coverslips, at a density of 5×10^4^ cells/well in a 48-well plate and cultured in the appropriate medium supplemented with 10% FBS. After 24 h, cells were shifted to serum-free medium for 18 h. Live MDA-MB-453, MDA-MB-231 and MCF-12A cells were labeled by indirect immunofluorescence as previously reported [28]. Briefly, unfixed cells were incubated with 2 µg/ml anti-Cdc37 antibody for 2 h. The cells were then washed, fixed in ice-cold acetone and labelled with Alexa488-conjugated secondary antibody.

### Preparation of cell lysates, Western blot analysis and Co-immunoprecipitation

Cells were grown in 25 cm^2^ flasks in culture medium supplemented with 10% FBS until sub-confluence, shifted in serum-free medium for 24 h and then exposed to anti-Cdc37 and/or mAb 4C5 for 16 h. Control cultures were grown either in culture medium alone or in culture medium containing 200 µg/ml of an IgG2a monoclonal antibody against the unrelated protein BM88 [35]. Excess non-bound antibody was then removed by washing with fresh medium. The cultures were immediately washed twice with ice-cold PBS and lysed as previously described [28].

Cellular fractionation was performed in cell cultures prepared and treated as described above, using the Compartmental protein extraction kit (Chemicon), according to the manufacturer's instructions.

Total and/or compartmental protein lysates were quantified, and equal amounts of total protein were subjected to SDS-PAGE followed by Western blot with the appropriate antibodies. Immunoreactive bands were detected using an ECL chemiluminescence reagent (Amersham Biosciences) and X-Omat AR film (Eastman Kodak Co.) as described by the manufacturers.

Co-immunoprecipitation was performed as previously described [25]. In brief, equal amounts of pre-cleared cellular lysates were incubated with the appropriate antibodies for 18 h at 4°C. The immunocomplexes were then incubated for 2 h at room temperature with protein G-Sepharose and washed three times with lysis buffer. Bound proteins were analyzed by gel electrophoresis followed by Western blot as described above. For all immunoprecipitation experiments, negative controls were performed using irrelevant IgGs.

### Antibody Internalization Assay

Antibody internalization experiments were performed as previously described [28]. Briefly, cells were incubated while in culture with 200 µg/ml anti-Cdc37 antibody for 16 h. The cells were then washed and fixed in cold acetone for 3 min. For detection of possible internalization of the antibody, cells were permeabilized with 0.1% Triton X-100 in PBS and subsequently incubated with Alexa488-conjugated secondary antibody (Molecular Probes). For all experiments, controls were performed using mAb 4C5 which is cell impermeable and the commercial anti-HSP90 which is internalized [27].

### RNA interference

Plasmid pLL3.7 with an insertion targeting Cdc37 was used. Cdc37 was knocked down using short hairpin RNAs. To this purpose the “WI siRNA Selection Program” from Whitehead Institute for Biomedical Research was used, to design and select the siRNA targeting Cdc37. In order to create a stem-loop structure, a spacer (TTCAAGAGA) was inserted into the sense and antisense sequences. DNA oligomers were annealed and cloned into the pLL3.7 plasmid. The insertion was confirmed by sequencing. MDA-MB 453 and MDA-MB 231 cells were grown at a density of 70% and then transfected with 3 µg of plasmid DNA with the inserted sequences or not (control), using the Xfect Transfection reagent from Clontech, according to manufacturers protocol. 72 hours after transfection, cells were collected, lysed and subjected to Western blot analysis. For immunofluorescence detection of Cdc37, cells were plated on poly-L-lysine coated coverslips and transfected with plasmid DNA as above. 72 hours after transfection, live cells were washed with PBS, incubated with anti-Cdc37 antibody for 2 hours, fixed with ice cold acetone and labeled with Alexa647-secondary antibody.

### Wound Healing Motility Assay

The assay was performed as previously described [28]. Briefly, MDA-MB-453 and MDA-MB-231 cells were plated in a 48-well plate at a density of 1.5×10^5^ cells/well and 5×10^4^ cells/well, respectively, and were left for 24 h in serum-containing medium, without further treatment. The medium was then changed to serum-free, and 16 h later, a cell-free area was generated by gently scratching the cell monolayer with a sterile yellow Gilson-pipette tip, thus resulting in the formation of a 1-mm-wide cell-free area. Immediately after scratching, the medium was replaced with fresh medium, or medium containing the anti-Cdc37 antibody. Control cultures were grown either in culture medium alone or in culture medium containing 200 µg/ml of an IgG2a monoclonal antibody against the unrelated protein BM88 [35].

Migration of breast cancer cells within the gap was monitored microscopically at given time intervals, using a Leica DM IL inverted microscope, equipped with a LEICA DM300 video camera connected to a computer. Inhibition of migration was estimated by acquiring and analyzing digital images, using the Image Pro Plus analysis software [36] and evaluated with two different ways depending on the cell line studied. More specifically for the MDA-MB-453 cells it was expressed as the percentage of distance covered by cells in control cultures, whereas for the MDA-MB-231 cells it was expressed as the percentage of cells observed within the gap of the wound in control cultures Statistical analysis was performed by using Student's *t* test.

## Results

### Cdc37 is expressed on the cell surface of MDA-MB-453 and MDA-MB-231 breast cancer cells

**Figure 1:**
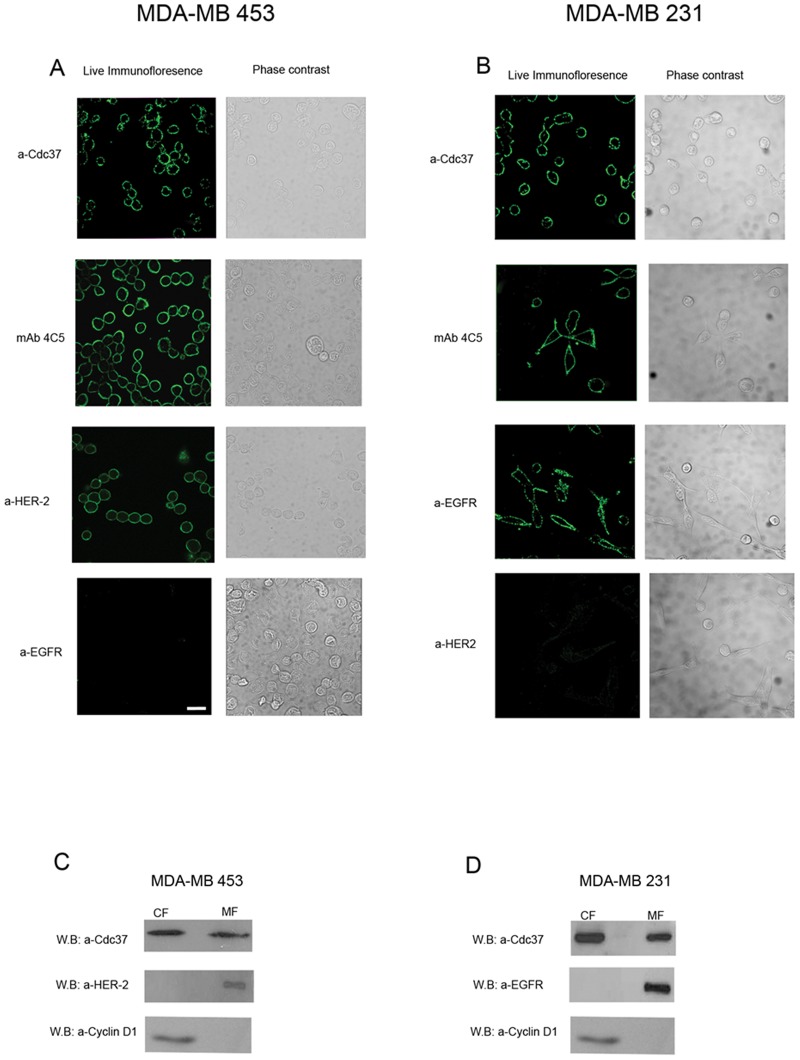
Cdc37 is localized on the cell surface of MDA-MB 453 and MDA-MB 231 breast cancer cells. **A**, Indirect immunofluorescence of live MDA-MB 453 cells using anti-Cdc37 antibody. The immunolabeling reveals the surface localization of Cdc37. Anti-HER2 and mAb 4C5 were used as positive controls. Anti –EGFR antibody was used as negative control. **B**, Indirect immunofluorescence of live MDA-MB 231 cells using anti-Cdc37 antibody. Anti-EGFR and mAb 4C5 were used as positive controls. Anti –HER2 antibodies was used as negative control. **C**, Cell fractionization of MDA-MB 453 cells followed by Western blot analysis confirmed the presence of Cdc37 in cell membrane fractions. Anti-HER2 antibodies were used as positive and negative controls in the membrane and cytosolic fractions respectively. **D**, Cell fractionization of MDA-MB 231 cells followed by western blot analysis confirmed the presence of Cdc37 on cell membrane fractions. Anti-EGFR antibodies were used as positive and negative controls in the membrane and cytosolic fractions respectively. In C and D antibody against the intracellular protein Cyclin D1 gave positive and negative immunostaining in the cytosolic and membrane fractions of both cell lines, respectively. CF, cytosolic fraction; MF, membrane fraction. Scale bar = 20 µm.

In order to examine the cell surface localization of Cdc37, unfixed MDA-MB-453 and MDA-MB-231 human breast cancer cells were incubated with the anti-Cdc37 antibody. The cells were then washed, fixed and labelled with Alexa488-conjugated secondary antibody. Thus the primary antibody had access only to the external surface of the cell. The observed immunostaining confirmed the cell surface localization of Cdc37 in both human breast cancer cell lines studied (Fig. 1). As positive control, mAb 4C5 which specifically binds to surface HSP90 whose presence has been previously shown in both cell lines [28] [26] was used. At this point it is of interest to note that immunofluorescence experiments showed, as expected, absence not only of Cdc37 but also of HSP90 on the surface of adult non cancerous MCF-12A cells (Fig. S1). Additional positive controls were applied, using antibodies against HER-2 and EGFR for the MDA-MB-453 and MDA-MB-231 cells, respectively. Finally antibodies against EGFR and HER2 were used as negative controls in MDA-MB-453 and MDA-MB-231 cells, respectively (Fig. 1A and B). The above results were confirmed by Western blot using membrane fractions derived from the two cell lines (Fig. 1C and D). As positive control anti-HER2 (Fig. 1C) and anti-EGFR (Fig. 1D) antibodies were used, in MDA-MB-453 and MDA-MB-231 membrane cell lysates, respectively. In the cytosolic fractions and as expected anti-Cdc37 gave positive immunostaining whereas the antibodies against the ErbB receptors were negative. Furthermore, the cytosolic fractions of both cell lines gave positive immunostaining when immunobloted with antibody against the intracellular protein Cyclin D1, whereas the membrane fractions were negative (Fig. 1C and D), thus confirming the purity of the fractions used here and in the immunoprecipitaion experiments described below.

**Figure 2:**
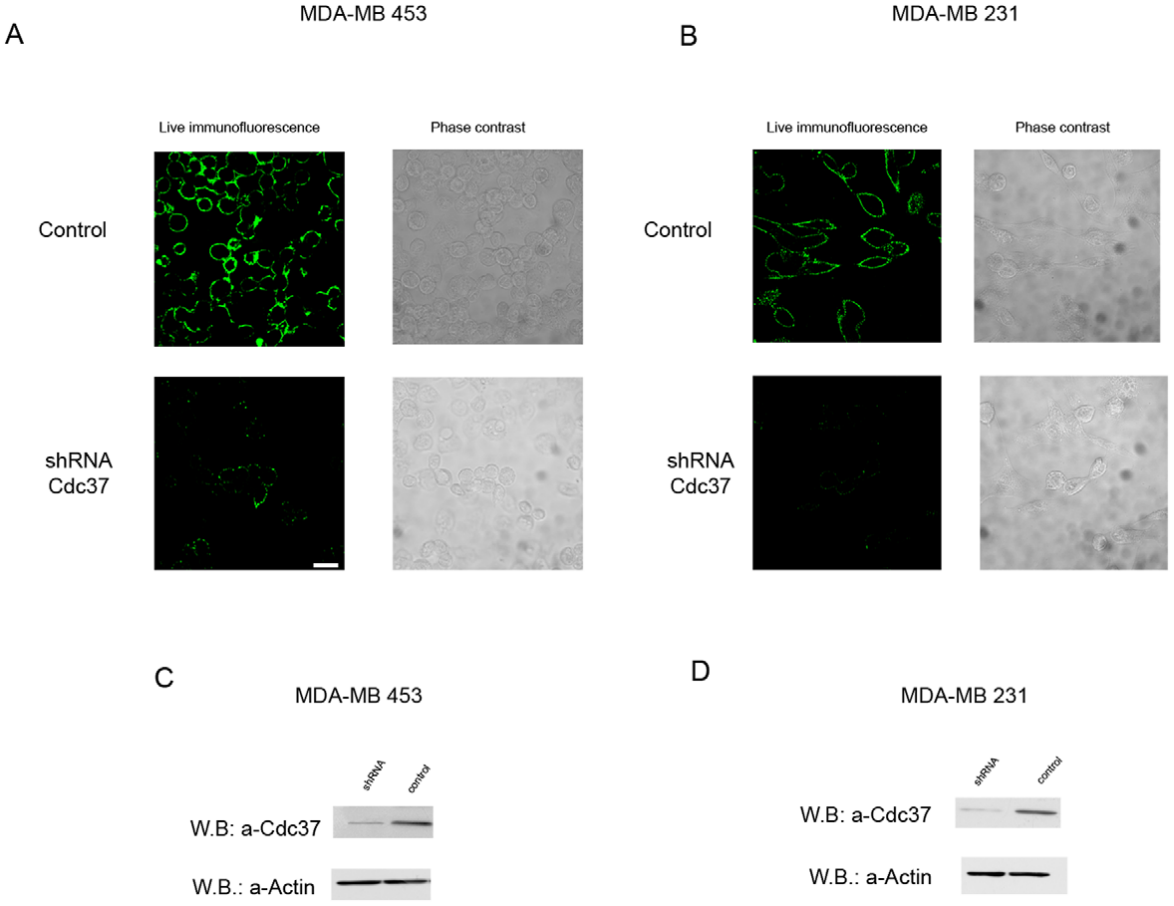
Specificity of the commercial anti-Cdc37 antibody and confirmation of the cell surface localization of Cdc37, using siRNA technology targeting Cdc37. A, B, immunofluorescence of live MDA-MB 453 and MDA-MB 231 cells respectively, using anti-Cdc37 antibody. Cells were transfected with siRNA targeting Cdc37 (shRNA Cdc37) or with plasmid DNA without any insertion (control). ShRNA Cdc37 transfected cells exhibit extremely low levels of surface immunofluorescence staining as compared to controls. Scale bar = 20 µm. C, D, Western blot analysis of total cell lysates derived from MDA-MB 453 and MDA-MB 231 cells respectively, using the anti-Cdc37 antibody, in cells transfected with siRNA targeting Cdc37 and control cells. The level of Cdc37 detected is significantly lower in both cell lines transfected with siRNA targeting Cdc37, when compared with control transfections.

In order to confirm the specificity of the commercial anti-Cdc37 antibody used in our studies, the siRNA technology was applied. More specifically, when MDA-MB-453 and MDA-MB-231cells transfected with siRNA targeting Cdc37 were immunostained as described above with the anti-Cdc37 antibody, an extremely weak labelling was observed when compared to cells tranfected with the control vector (Fig. 2 A and B). Similarly when western blot analysis using the anti-Cdc37 antibody was performed in total cell lysates derived from the above cells, a significantly decreased immunostaining was obtained in the lysates of the transfected with siRNA targeted against Cdc37 when compared to contols (Fig. 2C and D).

### The anti-Cdc37 antibody is cell-impermeable

**Figure 3:**
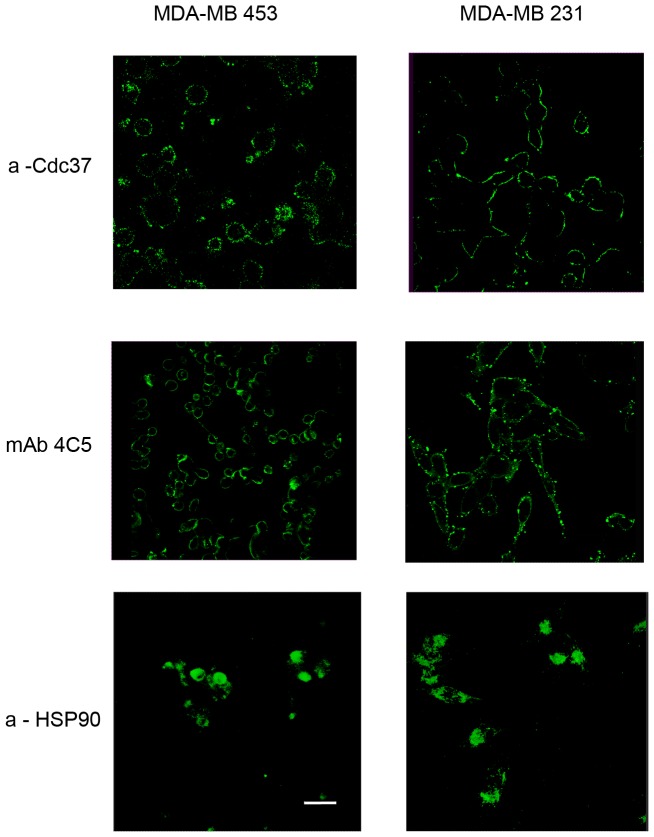
The anti-Cdc37 antibody is cell impermeable. Anti-Cdc37 antibody internalization was tested in both MDA-MB 453 and MDA-MB 231 cells. Live cells from the two cell lines were grown on coverslips and incubated at 37°C for 16 h with anti-Cdc37. MAb 4C5 and anti-HSP90 were used as positive and negative controls respectively. For detection of antibody internalization, cells were fixed and permeabilized as described in materials and methods and antibodies were detected by confocal microscopy using an Alexa 488 secondary antibody. No internalization of the anti-Cdc37 antibody was observed even in both cell lines. Scale bar = 20 µm.

The cell impermeable nature of the anti-Cdc37 used was examined by incubating the MDA-MB-453 and MDA-MB-231 cells while in culture with the antibody for 16 h. The cells were then carefully washed, and binding of the antibody was analyzed after fixation and cell permeabilization, using an Alexa488-conjugated secondary antibody. We observed that for both cell lines the anti-Cdc37 antibody was not internalized and remained bound on the cell surface (Fig. 3). MAb 4C5 and the polyclonal anti-HSP90 which have previously been shown to remain on the surface and be internalized respectively [27], were used as controls (Fig. 3). It should be noted that when the above mentioned cells were incubated with culture medium alone as a negative control, no immunostaining was obtained (data not shown).

### Surface Cdc37 is involved in breast cancer cell motility

**Figure 4:**
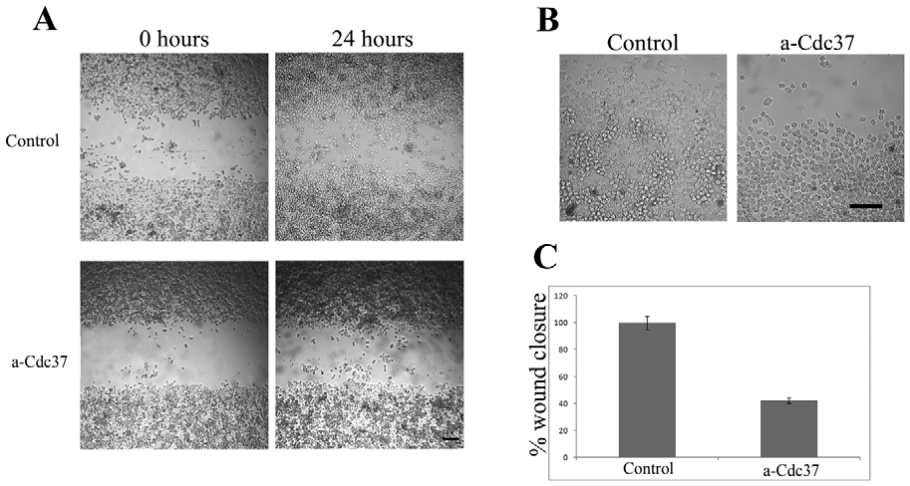
Cdc37 is involved in MDA-MB 453 cell invasion. **A**, Wound healing assay. Images obtained at 0 hours and 24 hours after scratch formation. Cells were let to migrate in control conditions or in the presence of 200 µg/ml of anti-Cdc37 antibody. **B**, At the end of the time point cells were stained with Trypan Blue. No significant cell death is observed in both cases. **C**, Quantitive effect of Cdc37 in cell migration. The addition of 200 µg/ml of anti-Cdc37 antibody in the cell culture medium resulted in a 57,7% inhibition of wound closure when compared to the control that was considered as 100% wound closure (P<0.005). Column is the average of three independent experiments ± SE. Within a single experiment each condition was tested in triplicate. Scale bars A = 165 µm, B = 80 µm.

**Figure 5:**
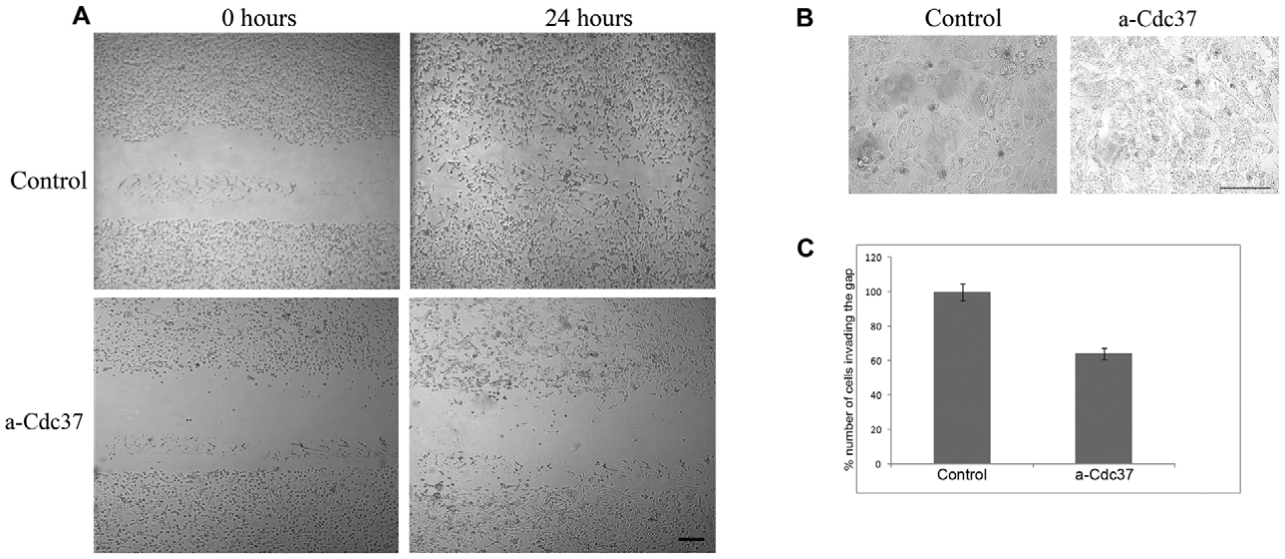
Cdc37 is involved in MDA-MB 231 cell invasion. **A**, Wound healing assay. Images obtained at 0 hours and 24 hours after scratch formation. Cells were let to migrate in control conditions as described in Materials and Methods or in the presence of 200 µg/ml of anti-Cdc37 antibody. **B**, At the end of the time point cells were stained with Trypan Blue. No significant cell death is observed in both cases. **C**, Quantitive effect of Cdc37 in cell migration. The addition of 200 µg/ml of anti-Cdc37 antibody in the cell culture medium resulted in 35,8% inhibition of cells invading the gap when compared to the control that was considered as 100% wound closure (P<0.005). Column is the average of three independent experiments ± SE. Within a single experiment each condition was tested in triplicate. Scale bars A = 165 µm, B = 80 µm.

Having established that the anti-Cdc37 antibody is not internalized but instead remains specifically bound to the cell-surface when incubated with living MDA-MB-453 and MDA-MB-231 cells, we next examined the possible involvement of surface Cdc37 on the motility of these breast cancer cells, using the wound healing assay. Control cultures were grown either in culture medium alone or in culture medium containing 200 µg/ml of an antibody against the unrelated protein BM88 [35]. It is important to note that no statistically significant difference was observed between the two types of controls used. The control value illustrated is the mean value of the two types of control. As shown in Fig. 4A, and Fig. 5A presence of the anti-Cdc37 antibody in the culture medium of both the MDA-MB-453 and MDA-MB-231 cells, resulted in a significantly slower rate of wound closure, when compared to controls cultures grown in culture medium alone. More specifically, a 57.7% and 35.8% inhibition of cancer cell motility was obtained when 200 µg/ml of anti-Cdc37 was included in the culture medium of MDA-MB-453 and MDA-MB-231 cells respectively (Figs. 4C and 5C). Trypan blue staining carried out at the end point of the wound healing assay, showed that the death rate of cells treated with the antibody was similar to that observed in the control cultures for both cell lines (Figs. 4B and 5B). This result further supports that the decreased cell motility observed in the presence of the anti-Cdc37 antibody which is not internalized is not caused by cell death but instead by the inhibition of the surface pool of Cdc37. At this point it is important to note that in this assay, the effect of the anti-Cdc37 antibody is directed towards cell invasion and not towards cell proliferation, since very low (<7%) BrdU incorporation was observed in both human breast cancer cell cultures treated as above with no apparent differences between the different experimental conditions (data not shown).

### Surface Cdc37 interacts with HSP90 in both breast cancer cell lines and with HER-2 and EGFR in MDA-MB-453 and MDA-MB-231cells respectively

**Figure 6:**
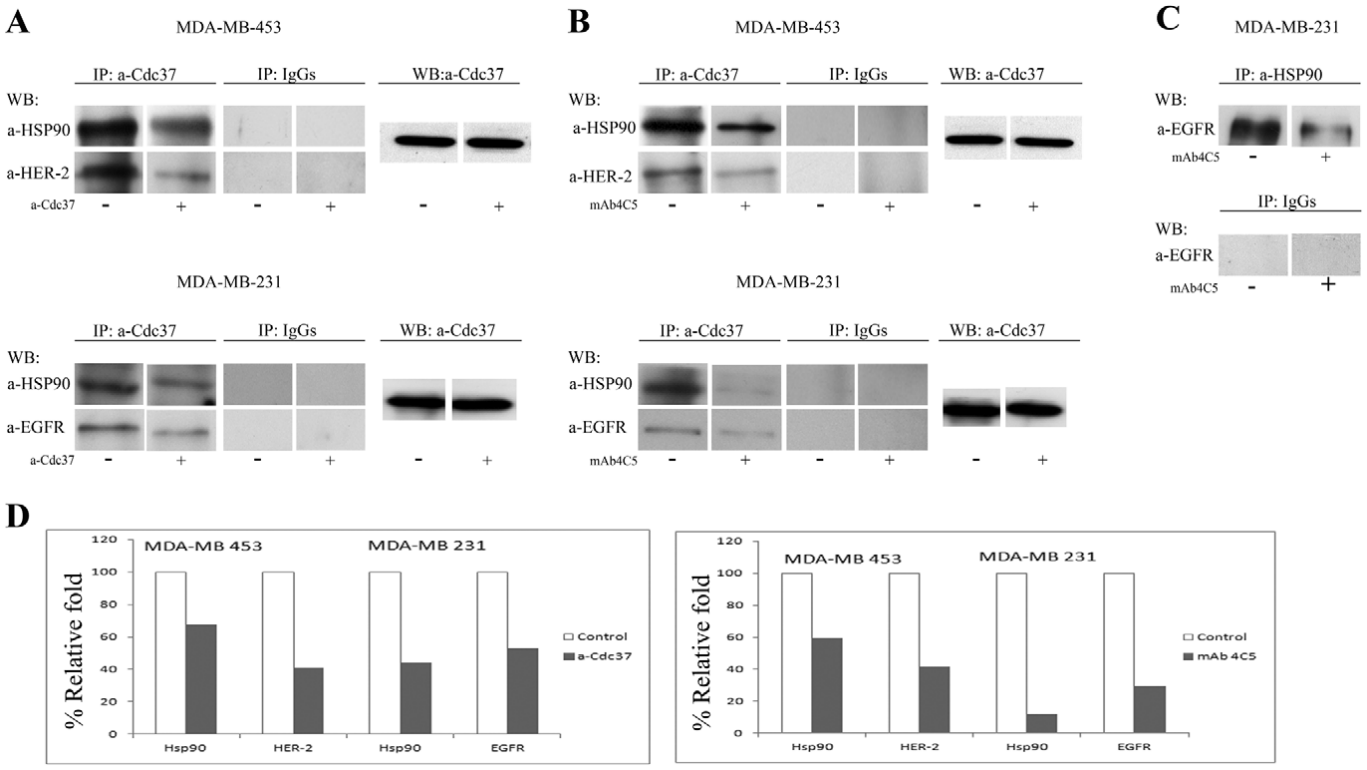
Cell surface interaction of Cdc37 and HSP90. A, Membrane protein fractions from MDA-MB 453 and MDA-MB 231 cells (control) were immunoprecipitaded with anti-Cdc37 antibody. Analysis of bound proteins by Western blot with anti HSP90 antibody and anti HER-2 and anti –EGFR antibodies respectively, revealed that Cdc37 interacts not only with HSP90 but also with both ErbB receptors studied. Presence of 200 µg/ml of the cell impermeable anti-Cdc37 for 16 h in the culture medium of the two cell lines followed by immunoprecipitation and Western blot analysis as above revealed that the anti-Cdc37 antibody disrupts the association of Cdc37 with HSP90 and the ErbB receptors in the two cell lines. **B**, MDA-MB 453 and MDA-MB 231 cells were treated or not with 200 µg/ml mAb 4C5 for 16 hours. Membrane protein fractions derived from these cultures were immunoprecipitaded with anti-Cdc37 followed by Western blot analysis using anti-HSP90 and anti-HER2 and anti EGFR antibodies respectively. **C**, Membrane protein fractions from MDA-MB 231 cells cultured in the absence or presence of 200 µg/ml of mAb 4C5 for 16 h were immunoprecipitated with the anti-EGFR antibody. Analysis of bound proteins by western blot with anti-HSP90 antibody revealed that surface HSP90 interacts with EGFR and that this interaction is disrupted by mAb 4C5. Irrelevant IgGs served as negative control for all the above experiments. **D**, Densitometry quantification of the observed reduced interactions in the presence of anti-Cdc37 in the culture medium of MDA-MB 453 and MDA-MB 231 cells resulted in a 33% and 59% decrease with HSP90 and HER-2 respectively regarding the MDA-MB 453 cells and in a 56% and 47% decrease with HSP90 and EGFR respectively regarding the MDA-MB 231 cells. Presence of mAb 4C5 in the culture medium of MDA-MB 453 and MDA-MB 231 cells resulted in a 60% and 58% decrease with HSP90 and HER-2 respectively regarding the MDA-MB 453 cells and in a 88% and 70% decrease with HSP90 and EGFR respectively regarding the MDA-MB 231 cells.

Taking into account the above shown involvement of surface Cdc37 in cancer cell invasion processes we next examined the possible interactions of this molecule with HSP90 and the ErbB kinase receptors. To this purpose we performed co-immunoprecipitation experiments using membrane fractions derived from the MDA-MB-453 and MDA-MB-231 cells cultured either in control medium as mentioned in Materials and Methods or in the presence of 200 µg/ml of anti-Cdc37 antibody for 16 h and immunoprecipitated with the anti-Cdc37 antibody. Western blot analysis using anti-HSP90, anti-HER2 and anti-EGFR antibodies respectively, revealed that the anti-Cdc37 antibody specifically disrupts the surface interaction of Cdc37 with HSP90 and both receptors in the two cell lines studied (Fig. 6A) since a reduced amount of these molecules was observed in the treated cultures when compared to the controls. At this point it is important to note that the observed disruption confirms the cell surface localization of the molecular interactions studied, since as shown above, the anti-Cdc37 antibody is not internalized and remains bound to the cell surface when included in the culture medium.

### MAb 4C5 specifically disrupts the surface interaction of Cdc37 with HSP90 and the ErbB receptors

Having established that similarly to its intracellular counterpart, surface Cdc37 is physically associated with surface HSP90, we next sought to investigate the effect of the function blocking anti-HSP90 monoclonal antibody, mAb 4C5, in this interaction. To this end, membrane fractions derived from MDA-MB-453 and MDA-MB-231 cultures that were treated with 200 µg/ml of mAb 4C5 for 16 h were immunoprecipitated with the anti-Cdc37 antibody. Western blot analysis using anti- HSP90 revealed that mAb 4C5 specifically disrupts the surface interaction of Cdc37 with HSP90 in both cancer cell lines used (Fig. 6B). More specifically the anti-Cdc37 antibody co-immunoprecipitated lower levels of HSP90 in the mAb 4C5 treated cultures when compared to the controls.

We have previously shown using mAb 4C5 that surface HSP90 specifically interacts with the extracellular domain of HER-2 in MDA-MB-453 cells [28].In the present work and in order to further examine the mode of action of surface Cdc37 in the two cell lines studied, it was necessary to first investigate the possible interaction of the surface pool of HSP90 with EGFR. To this end, membrane fractions derived from MDA-MB-231 cultures treated in the absence or in the presence of 200 µg/ml mAb 4C5 for 16 h, were immunoprecipitated with the anti-HSP90 antibody and immunoblotted with anti-EGFR. As shown in Fig. 6C EGFR specifically interacts with HSP90. The observed reduction in the presence of the cell impermeable mAb 4C5 indicates the cell surface localization of this interaction Having shown the association of surface HSP90 and EGFR and additionally that Cdc37 interacts not only with HSP90 but also with HER2 and EGFR we next sought to investigate the possible effect of mAb 4C5 on the co-chaperone's interaction with the ErbB receptors. Accordingly membrane fraction derived from MDA-MB-453 and MDA-MB-231 cultures treated or not with 200 µg/ml mAb 4C5 for 16 h were immunoprecipitated with the anti-Cdc37 antibody. Western blot analysis using anti-HER2 and anti-EGFR antibodies respectively, revealed that mAb 4C5 specifically disrupts the surface interaction of Cdc37 with both receptor molecules since lower levels of the ErbB receptors were observed in the mAb 4C5 treated cultures (Fig. 6B).

## Discussion

In the present work we demonstrate that the co-chaperone Cdc37 is localized on the surface of MDA-MB-453 and MDA-MB-231 breast cancer cells, where it is necessary for the motility of these cells and similarly to its intracellular counterpart it specifically interacts with the molecular chaperone HSP90. Moreover our findings show that this surface pool of Cdc37 directly interacts with members of the ErbB family of growth factor receptors possibly acting as a co-factor in HSP90 extracellular chaperoning activities implicated in cancer cell invasion processes and that the anti-HSP90 antibody mAb 4C5 disrupts these interactions.

Cell surface localization of Cdc37 was demonstrated by both immunocytochemistry on live MDA-MB-453 and MDA-MB-231 breast cancer cells and western blot using membrane fraction lysates derived from these cell lines.

The inhibition of MDA-MB-453 and MDA-MB-231 breast cancer cell motility, by the anti-Cdc37 antibody was shown using the wound healing assay. Presence of this antibody in the culture medium significantly reduced the motility rate of both cell lines studied. At this point it is interesting to note that when the anti-HSP90 antibody mAb 4C5 and the anti-Cdc37 antibody were included either separately or combined in the culture medium of the above cells no statistically significant differences were observed in the wound closures. This is not surprising since mAb 4C5 and anti-Cdc37 are targeted against two different molecules involved in the cell motility process (data not shown).

To assess the participation of cell surface and not intracellular Cdc37 in the motility process of the cancer cells studied, internalization of the anti-Cdc37 antibody was examined. Interestingly, the antibody remained bound to the cell surface of both MDA-MB-453 and MDA-MB-231 cells, even after 16 h in culture and was not internalized indicating that the previously mentioned inhibitory effect of the anti-Cdc37 antibody is due to binding of the antibody to the cell surface pool of Cdc37.

It is well established that intracellular Cdc37 acts as co-chaperone of HSP90 by targeting protein kinases to the chaperone machinery and thus contributing to their activation. Taking this into account together with the above mentioned results we next examined the possible association of cell surface Cdc37 with the surface pools of HSP90 and the ErbB receptors. Indeed immunoprecipitation experiments using MDA-MB-453 and MDA-MB-231 cell lysates showed that Cdc37 interacts not only with HSP90 but also with HER2 and EGFR respectively. Disruption of these interactions when the cell impermeable anti-Cdc37 was included in the culture medium of the cell lines studied, further confirmed the cell surface localization of the interacting molecules. Our results in combination with previously reported data showing contribution of cell surface HSP90 in cancer cell invasion [28] suggest that surface Cdc37 participates in cancer cell invasion by acting similarly to its intracellular counterpart as co-chaperone to surface HSP90 in association with the protein kinase receptors.

We have previously shown that participation of surface HSP90 in cancer cell invasion processes occurs through its interaction with the extracellular domain of HER-2 in MDA-MB-453 cells, and that this interaction is disrupted by a the cell impermeable anti-HSP90 antibody mAb 4C5 [28]. As a follow up of these results in the present work we investigated the involvement of surface HSP90 in the invasion processes of MDA-MB-231 cells and the possible association of this chaperone with EGFR. Immunoprecipitation experiments in the presence or absence of mAb 4C5 in the culture medium of MDA-MB-231 cells revealed that mAb 4C5 strongly inhibits the invasive capacity of these cells (data not shown), and that it disrupts interaction between surface HSP90 and EGFR. To our knowledge this is the first report of a surface interaction between HSP90 and EGFR.

Taking into account all the above results we next examined the possible effect of mAb 4C5 on the interactions of Cdc37 with HSP90 and the ErbB receptors. Indeed, immunoprecipitation experiments in MDA-MB-453 and MDA-MB-231 membrane fractions of cells cultured in the absence or presence of mAb 4C5 revealed that this antibody significantly decreased the amount not only of HSP90 but interestingly also of HER-2 and EGFR respectively in the Cdc37-immunoprecipitated lysates, indicating that the formation and/or stabilization of the Cdc37/HSP90/kinase receptor complex is disrupted by mAb 4C5. In the above mentioned complex, HSP90 interacts simultaneously with Cdc37 via its N- terminal region [5] whilst a region within the N-terminal and middle domain is associated with the kinase client protein [37]. Moreover Cdc37 is associated with the catalytic domain of the kinase client via its N-terminal domain [5]. Taking into account these reports, the following interpretation of our results is largely at the level of speculation. It is conceivable that mAb 4C5 may bind either a) to the N terminal of HSP90 and thus inhibit the HSP90 –Cdc37 interaction which in turn may induce conformational changes to the two molecules that prevent their association with the kinase receptors or b) to a region within the N terminal and middle domain of the chaperone and thus inhibit the HSP90-kinase receptor interaction which may result in conformational changes of these molecules that prevent Cdc37 association with HSP90 and the ErbB receptors. This last scenario is supported by very recent data [38] demonstrating that a 115-aa fragment within the linker region and middle domain of secreted HSP90 acts as a pro-motility wound healing agent in mice. In any case our data strongly indicate that the conformational state of the molecules in the HSP90/Cdc37/kinase receptor complex is of great importance for their effective interactions.

In conclusion in the present work we demonstrate the cell surface localization of Cdc37 and its participation in invasion processes of breast cancer cells. Moreover we show that this surface pool of Cdc37 is associated with HSP90 and the ErbB kinase receptors HER2 and EGFR, thus indicating that it acts similarly to its intracellular counterpart as co-chaperone in the HSP90 machinery. Finally we show that the cell impermeable anti-HSP90 mAb 4C5 which has been previously shown to inhibit cancer cell invasion and metastasis [27], [28], [29] has the capacity to impair all the molecular interaction in the Cdc37/HSP90/kinase receptor complex. Our study reinforces existing reports regarding the oncogenic role of Cdc37 and its value as a target for cancer therapy.

## Supporting Information

Figure S1 Cdc37 and HSP90 are absent from the cell surface of adult non cancerous MCF-12A cells. Indirect immunofluorescence of live MCF-12A cells using anti-Cdc37 antibody (**A**) and anti-HSP90 antibody (**B**), revealed absence of these molecules from the cell surface. In contrast, expression of Cdc37 (**A**) and HSP90 (**B**) proteins is very intense in the cytoplasm of these cells. Scale bar = 20 µm.
(TIF)
