# Supplementary figures and images for: Cell Surface Cdc37 Participates in Extracellular HSP90 Mediated Cancer Cell Invasion
Source: PLoS One. 2012 Aug 17;7(8):e42722. doi: 10.1371/journal.pone.0042722 (PMC3422348; doi:10.1371/journal.pone.0042722)

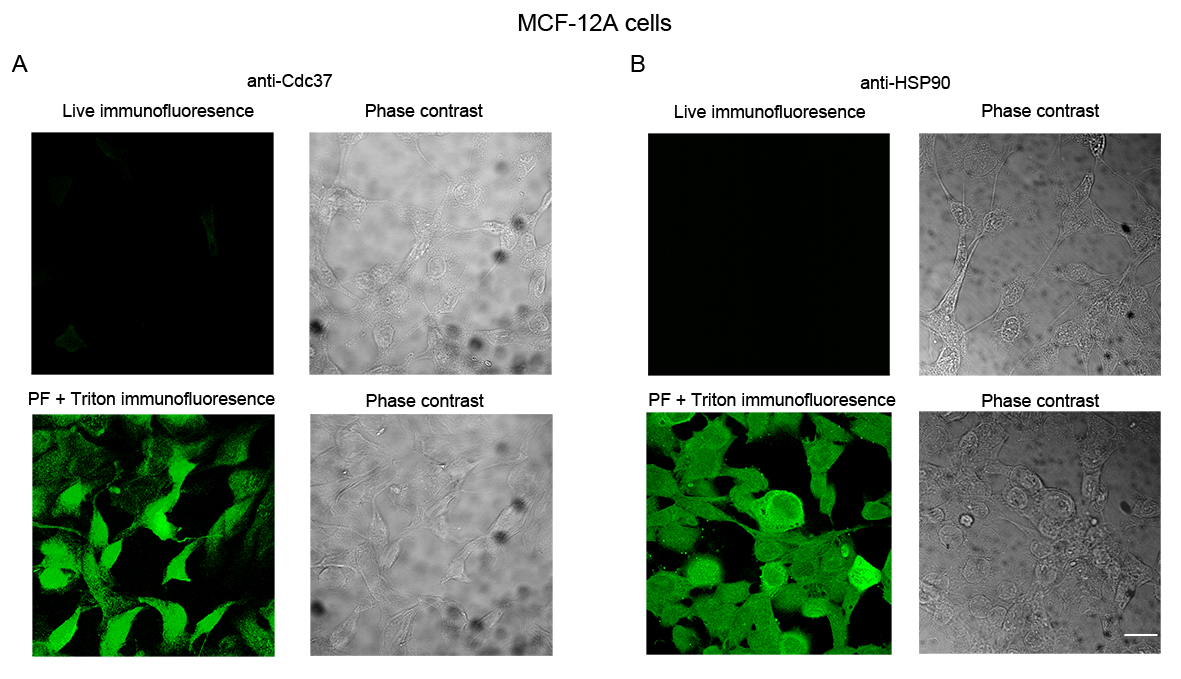

Supplement: Figure S1 — Cdc37 and HSP90 are absent from the cell surface of adult non cancerous MCF-12A cells. Indirect immunofluorescence of live MCF-12A cells using anti-Cdc37 antibody (A) and anti-HSP90 antibody (B), revealed absence of these molecules from the cell surface. In contrast, expression of Cdc37 (A) and HSP90 (B) proteins is very intense in the cytoplasm of these cells. Scale bar = 20 µm. (TIF) [file pone.0042722.s001.tif]
